# Laparoscopic D3 lymph node dissection with left colic artery and first sigmoid artery preservation in rectal cancer

**DOI:** 10.1186/s12957-023-02964-4

**Published:** 2023-03-06

**Authors:** Xing Huang, Zhigang Xiao, Zhongcheng Huang, Dan Li

**Affiliations:** The First Department of General Surgery (Department of Colorectal and Anal Surgery), Hunan Provincial People’s Hospital (The First Affiliated Hospital of Hunan Normal University), No. 61 Jiefang West Road, Changsha, Hunan China

**Keywords:** Rectal cancer surgery, Anastomotic leakage, D3 lymph node dissection, Left colic artery (LCA), Sigmoid artery (SA)

## Abstract

**Background:**

D3 lymph node dissection with left colic artery (LCA) preservation in rectal cancer surgery seems to have little effect on reducing postoperative anastomotic leakage. So we first propose D3 lymph node dissection with LCA and first sigmoid artery (SA) preservation. This novel procedure deserves further study.

**Methods:**

Rectal cancer patients who underwent laparoscopic D3 lymph node dissection with LCA preservation or with LCA and first SA preservation between January 2017 and January 2020 were retrospectively assessed. The patients were categorized into two groups: the preservation of the LCA group and the preservation of the LCA and first SA group. A 1:1 propensity score-matched analysis was performed to decrease confounding.

**Results:**

Propensity score matching yielded 56 patients in each group from the eligible patients. The rate of postoperative anastomotic leakage in the preservation of the LCA and first SA group was significantly lower than that in the LCA preservation group (7.1% vs. 0%, *P*=0.040). No significant differences were observed in operation time, length of hospital stay, estimated blood loss, length of distal margin, lymph node retrieval, apical lymph node retrieval, and complications. A survival analysis showed patients’ 3-year disease-free survival (DFS) rates of group 1 and group 2 were 81.8% and 83.5% (*P*=0.595), respectively.

**Conclusion:**

D3 lymph node dissection with LCA and first SA preservation for rectal cancer may help reduce the incidence of anastomotic leakage without compromising oncological outcomes compare with D3 lymph node dissection with LCA preservation alone.

**Supplementary Information:**

The online version contains supplementary material available at 10.1186/s12957-023-02964-4.

## Introduction

**Fig. 1:**
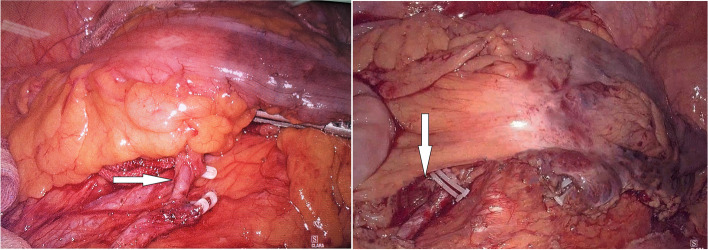
Proximal colonic ischemia in rectal cancer surgery with the LCA preservation (arrows)

Anastomotic leakage is still a fatal complication, with an incidence of about 10–15% [1, 2]. One of the most crucial risk factors is anastomotic perfusion, which is also one of the few aspects that surgeons can optimize for rectal cancer patients [2–4]. Therefore, the preservation of more branches of the inferior mesenteric artery (IMA) in rectal cancer surgery should be an optimal strategy. Some studies [5–7] have also suggested blood flow of the proximal bowel, and the anastomosis could be improved by left colic artery (LCA) preservation. At the same time, although with a low metastatic rate, apical lymph node metastasis is an important prognostic factor for rectal cancer patients [8–11]. The LCA preservation has become more and more popular in rectal cancer surgery. However, some other studies [12–15] have suggested that compared with LCA non-preservation, it has very little effect on reducing postoperative anastomotic leakage. We do encounter situations of proximal colonic ischemia sometimes in rectal cancer surgery with LCA preservation (Fig. 1). Possible reasons include a weak marginal artery or anatomically vulnerable to ischemia, such as Griffith’s point and Sudeck’s critical point. For these cases, even if the LCA is preserved in rectal cancer surgery, it has little effect on increasing the blood supply of the anastomosis. The preservation of the sigmoid artery (SA) may be more important for these cases than that of LCA. Therefore, we first propose a novel procedure, D3 lymph node dissection with LCA and first SA preservation in rectal cancer surgery [16]. Compared with the LCA preservation alone, the main supplying arteries (LCA and SA) of patients in our procedure are preserved. From the perspective of anatomical theory, this procedure may bring different results.

## Materials and methods

### Data

**Fig. 2:**
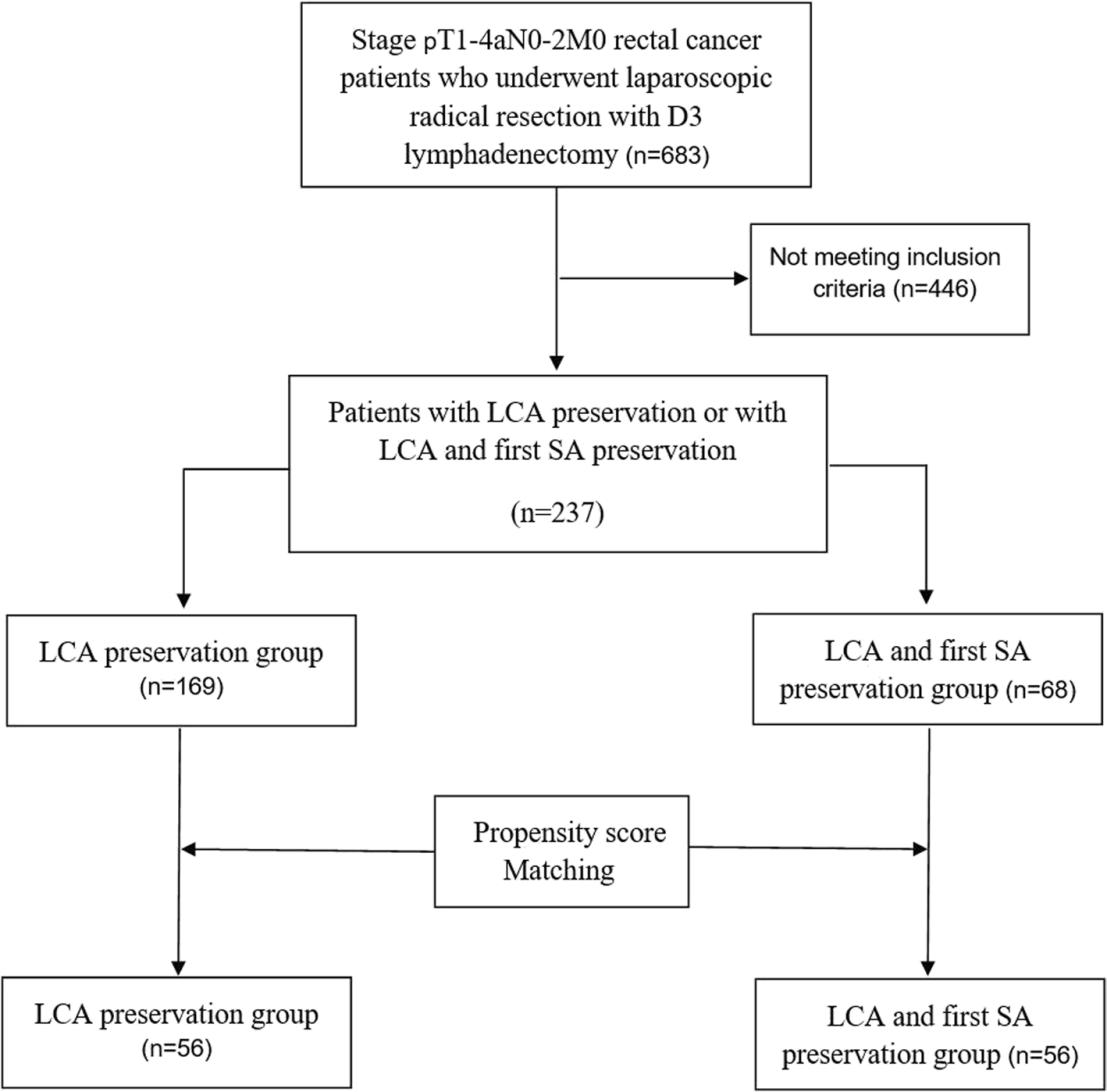
The study flowchart

Rectal cancer patients who underwent laparoscopic radical resection with D3 lymphadenectomy by skilled surgeons between January 2017 and January 2020 at Hunan Provincial People’s Hospital were retrospectively assessed. The skilled surgeons perform at least 100 laparoscopic colorectal resections every year and have completed their learning curve in laparoscopic colorectal operations. A total of 683 rectal cancer (distal tumor margin distance from the anal verge ≤15cm) patients’ medical records (Stage pT1-4aN0-2M0) who had undergone the radical resection with D3 lymphadenectomy were retrospectively reviewed. Among them, 237 patients met the inclusion criteria. After 1:1 propensity score matching, 56 patients were matched into an LCA preservation group and 56 patients into an LCA and first SA preservation group. The inclusion criteria included the following: (1) 18 years of age and over, (2) laparoscopic D3 lymph node dissection with LCA preservation or with LCA and first SA preservation, and (3) American Society of Anesthesiologists (ASA) grade less than or equal to III. The exclusion criteria included the following: (1) incomplete data, (2) synchronous colorectal carcinoma, (3) emergency surgery, (4) primary tumor that was not R0-resected, and (5) history of colon or rectal segmental resections. Figure 2 shows the study flow chart. This study was approved by the ethics committee of Hunan Provincial People’s Hospital. The study was performed in accordance with the ethical standards laid down in the 1964 Declaration of Helsinki and its later amendments or comparable ethical standards.

Group 1 included 56 patients with LCA preservation, Group 2 included 56 patients with LCA and first SA preservation. The clinical characteristics (included age at surgery, gender, BMI, ASA score, tumor size, tumor location, tumor stage(UICC), loop ileostomy, and neoadjuvant therapy) after PSM between these two groups were compared. Similarly, the surgical and pathological outcomes (included operation time, length of hospital stay, estimated blood loss, length of distal margin, lymph node retrieval, apical lymph node retrieval, complications, and anastomotic leakage (grade B and C)) between these two groups were compared. At last, the survival curves of the two groups for DFS were drawn accordingly.

### Surgical procedure

**Fig. 3:**
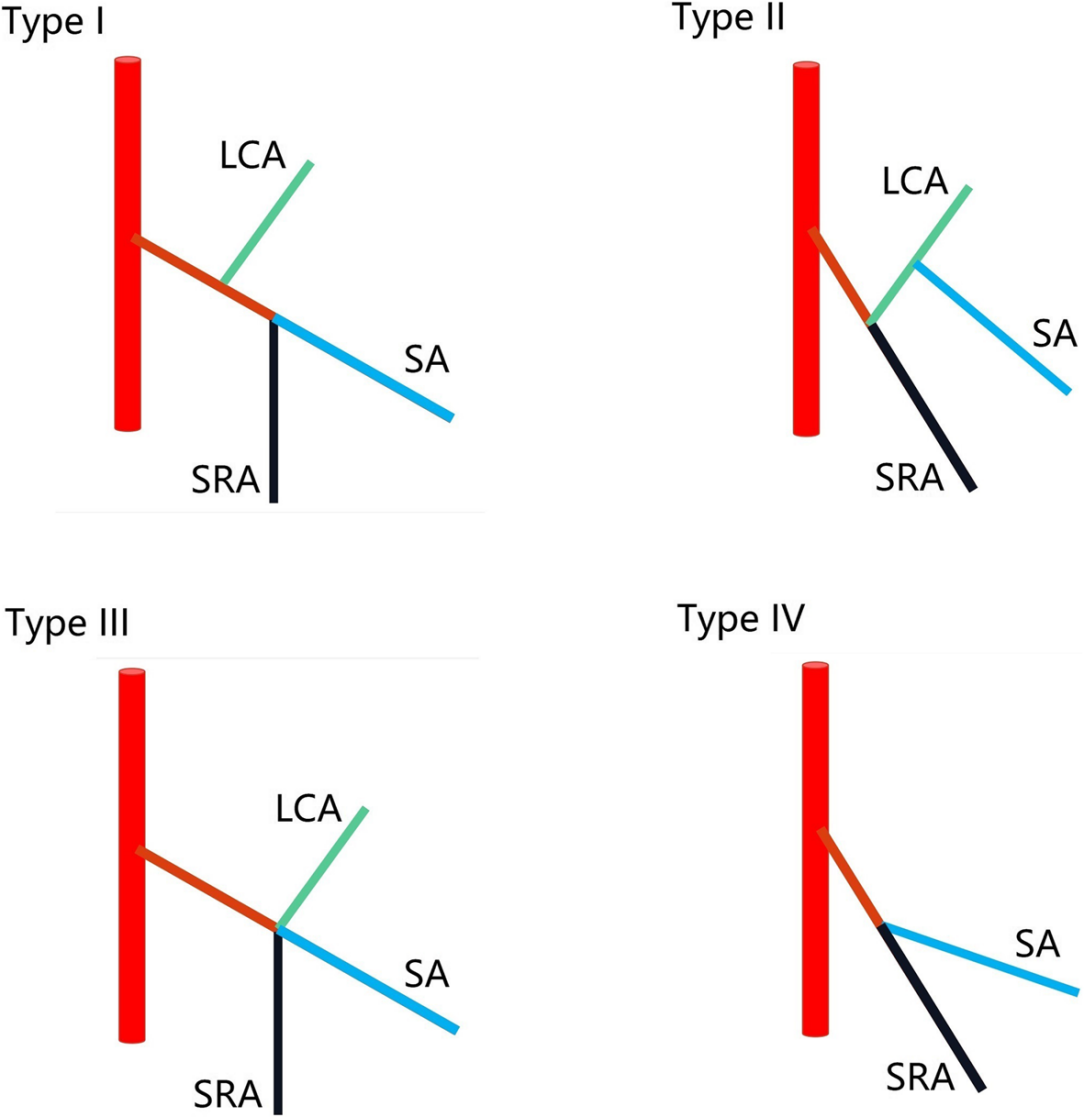
Four common types of IMA from the Yada classification (type I: LCA and SA emanate from IMA independently; type II: LCA and SA co-trunk; type III: LCA, SA, and SRA emanate from the same point; type IV: LCA is absent)

**Fig. 4:**
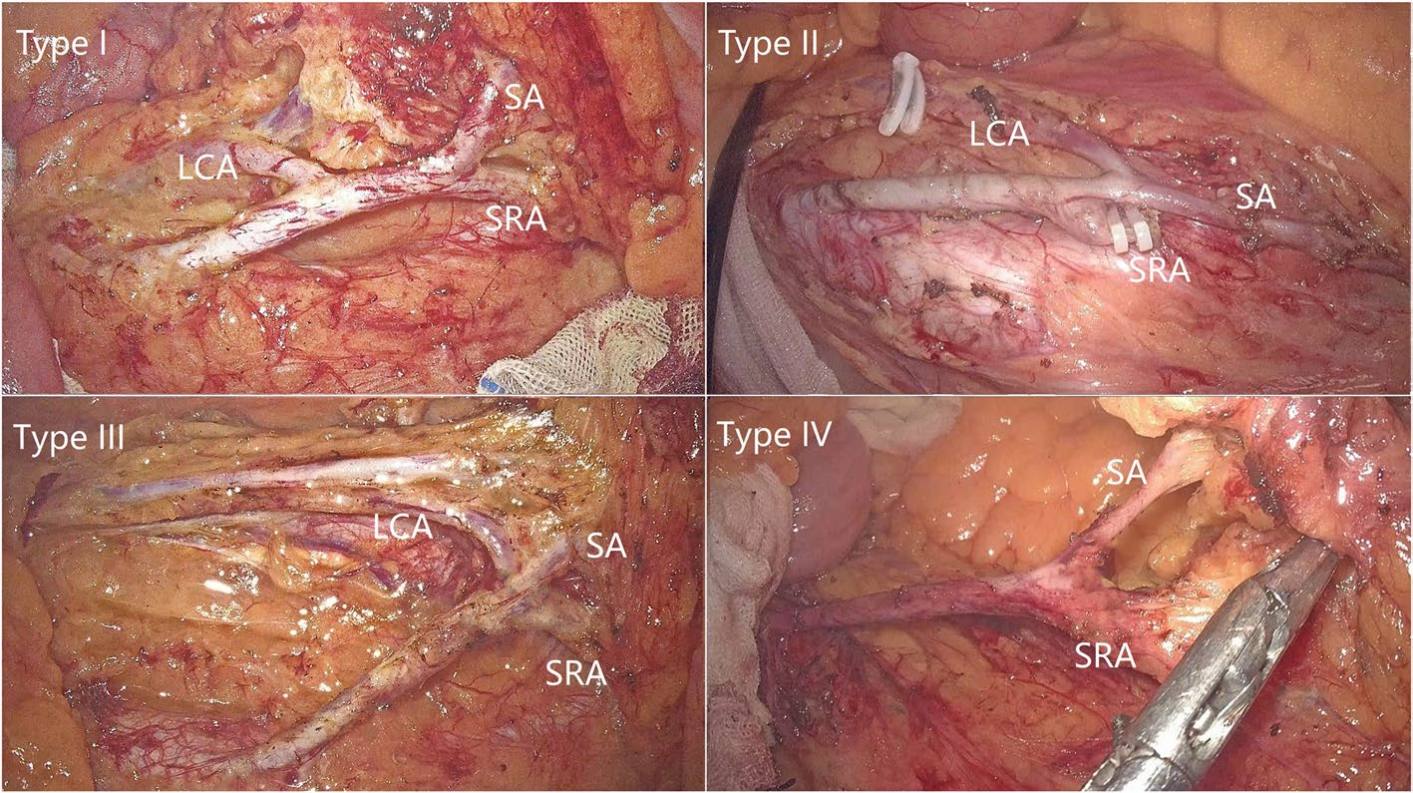
Laparoscopic views of four common types of IMA (type I: LCA and SA emanate from IMA independently; type II: LCA and SA co-trunk; type III: LCA, SA, and SRA emanate from the same point; type IV: LCA is absent)

**Fig. 5:**
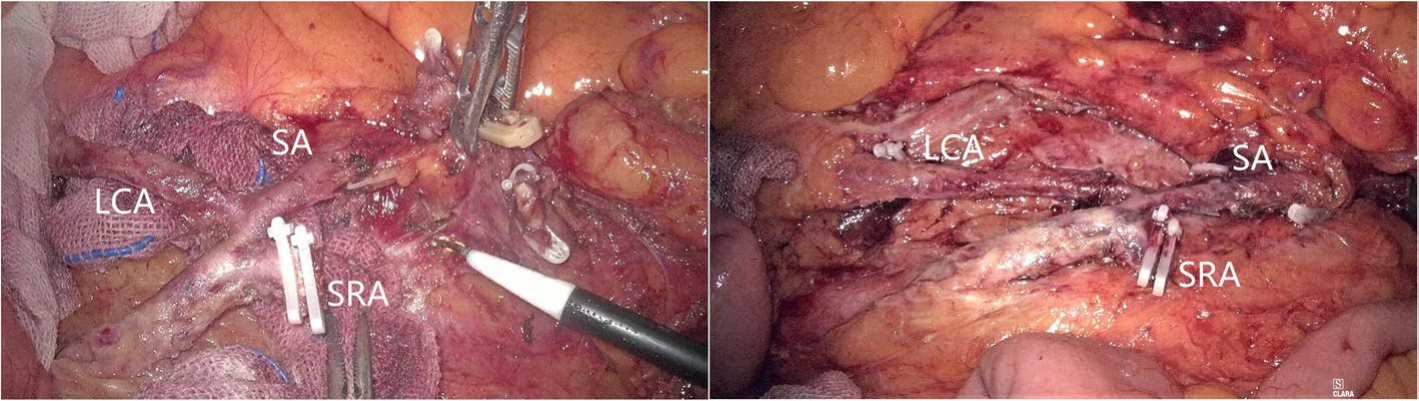
Laparoscopic view showing diffuse mesenteric edema and fibrosis from a patient who received neoadjuvant therapy, while the LCA and the first SA are well preserved

In recent years, we have focused on the surgical technique of more branches of the IMA preservation in rectal cancer surgery with D3 lymph node dissection. At present, Yada’s classification [17, 18] published in 1997 is the most widely recognized classification for IMA (Fig. 3). Our procedure could be carried out smoothly for most patients (Fig. 4), even for patients who received neoadjuvant therapy (Fig. 5). Here, we demonstrate D3 lymph node dissection with the LCA and the SA preservation in rectal cancer patients with or without neoadjuvant therapy (video). In addition, we need to point out that not all patients are suitable for our procedure, sometimes, preservation of the first SA may lead to anastomotic tension, even lead to anastomotic failure.

Based on the Chinese BMI classification, BMIs are classified as underweight (BMI < 18.5 kg/m^2^), normal (BMI 18.5–23.9 kg/m^2^), and overweight and obese (BMI ≥ 24 kg/m^2^). Postoperative complications are graded according to the Clavien-Dindo classification, and we group complications into major (Clavien-Dindo III–V) and minor (Clavien-Dindo I–II) complications. Classification of anastomotic leakage is graded according to the International Study Group of Rectal Cancer. Grade A leakage results in no change in patients’ management, whereas Grade B leakage requires active therapeutic intervention but is manageable without re-laparotomy. Grade C leakage requires re-laparotomy. The disease-free survival (DFS) in this study is defined as the time from operation to recurrence, death, or to the last follow-up date. The postoperative follow-up includes a physical examination, serum CEA, CA19-9, chest X-ray or CT, coloscopy, abdominal CT, and positron emission tomography scanning, if available. Of the 112 patients, only 7 (6.25%) receive neoadjuvant therapy. Recurrence is determined by the above examinations or by histologic analysis. The last follow-up is performed in November 2022.

### Statistical analysis

Continuous variables were described as the mean ± standard deviation, chi-squared test, and independent-sample *t* test were used for statistical analysis. The Kaplan-Meier method was used for drawing the survival curves, and comparison of survival between the groups was performed by the log-rank test. All statistical analyses were performed using the SPSS program (Statistical Product and Service Solution 20 for Windows; SPSS Inc., Chicago, IL). The caliper of the 1:1 propensity score-matched analysis was calculated to be 0.03. A value of *P* < 0.05 was considered as the limit for statistical significance.

## Results

### Patient characteristics

The patients’ baseline characteristics of the two groups after PSM are outlined in Table 1. There were no statistically significant differences between the two groups. The patients’ surgical and pathological outcomes of the two groups are outlined in Table 2. There was a significant difference in postoperative anastomotic leakage (grades B and C) between the two groups.

**Table 1:** Patients’ baseline characteristics after PSM

Total	Group 1	Group 2	*P* value
	56	56	
Age at surgery (years)			
<60	20	17	0.547
≥60	36	39	
Gender			
Female	21	27	0.252
Male	35	29	
BMI (kg/m^2^)			
Underweight	10	8	0.617
Normal	33	38	
Overweight and obese	13	10	
ASA score			
1	5	7	0.706
2	37	33	
3	14	16	
Tumor size (cm)			
<4	31	33	0.703
≥4	25	23	
Tumor location (cm)			
<10	31	23	0.130
≥10	25	33	
Tumor stage(UICC)			
I	9	9	0.825
II	21	24	
III	26	23	
Loop ileostomy			
No	50	48	0.568
Yes	6	8	
Neoadjuvant therapy			
No	52	53	0.696
Yes	4	3	

BMI classifications using Chinese BMI classification. Tumor location according to distal tumor margin distance from the anal verge

**Table 2:** Surgical and pathological outcomes of these patients

Total	Group 1	Group 2	*P* value
	56	56	
Operative time(min)	171.7±24.7	175.5±23.7	0.408
Length of hospital stay(day)	12.0±4.9	11.2±2.8	0.305
Estimated blood loss(mL)	24.6±11.1	26.6±13.5	0.403
Length of distal margin(cm)	2.5±0.6	2.3±0.5	0.093
Lymph node retrieval	17.6±7.2	17.0±6.4	0.668
Apical lymph node retrieval	1.1±1.3	1.1±1.2	0.941
Complications			
I-II	44	48	0.324
III-V	12	8	
Anastomotic leakage(grades B and C)			
No	51	56	0.040
Yes	4	0	

Classification of surgical complications using Clavien-Dindo classification. Classification of anastomotic leakage according to the International Study Group of Rectal Cancer

### Results of the follow-up

**Fig. 6:**
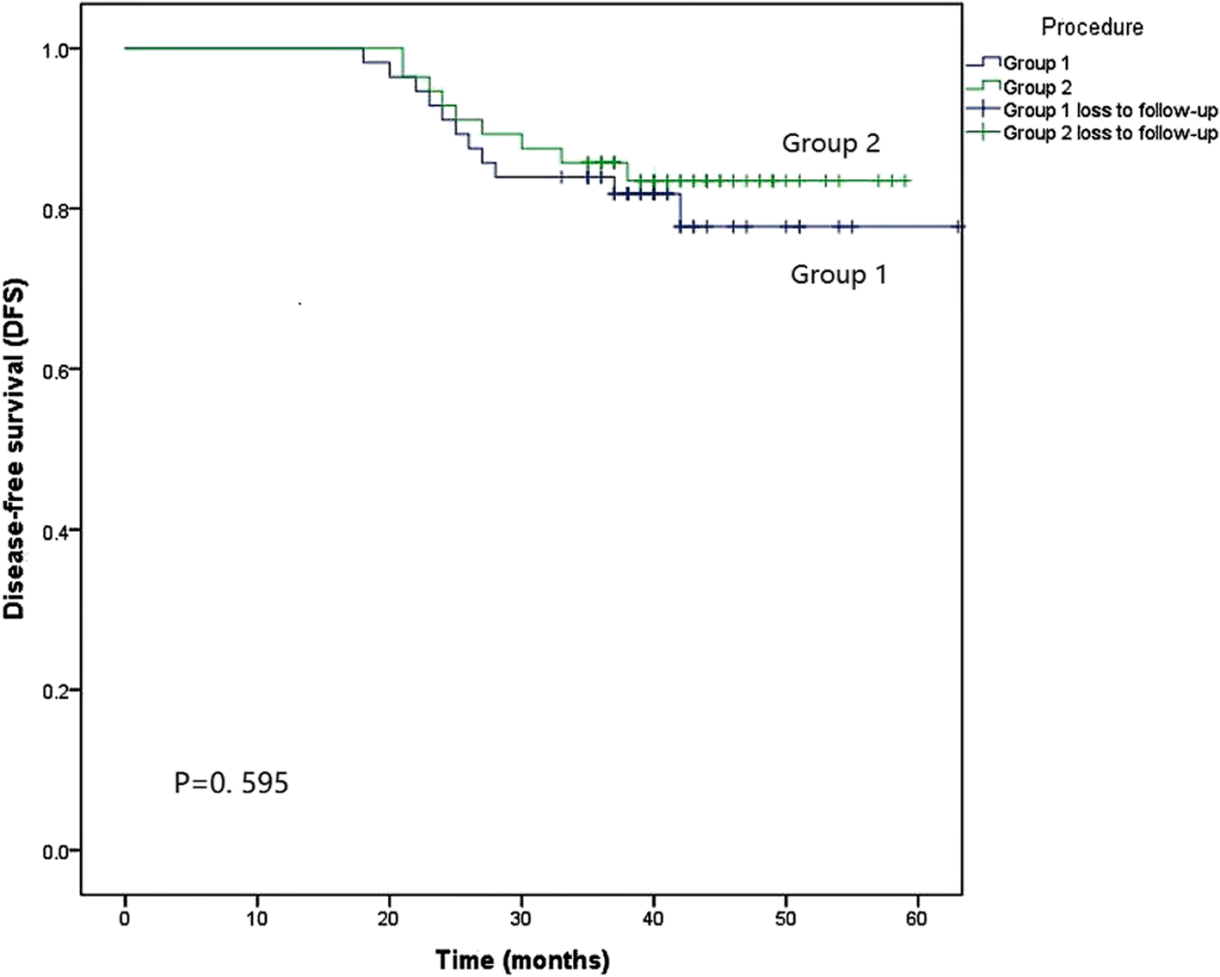
Survival curves of groups 1 and 2 for DFS

With a median follow-up period of 40 months (range 18 to 70), the patients’ 3-year DFS rates of group 1 and group 2 were 81.8% and 83.5%, respectively. There was no significant difference in DFS between the two groups (*P*=0.595). The survival curves of group 1 and group 2 for DFS are in Fig. 6.

## Discussion

High tie (HT) and low tie (LT) of the IMA are two hot topics in colorectal cancer surgery. The former is ligation at the root of the IMA and does not retain the LCA, and the latter is ligation over the LCA branch of the IMA and retains the LCA. According to anatomy, HT may have a better oncological outcome due to a more extended apical lymph node dissection, while LT may have a better anastomotic blood supply due to LCA preservation. “HT or LT” has been a hot research topic in recent years [14, 19–22]. Although many previous studies believe that the two ligation methods had little effect on the oncological outcomes, we still could not deny the importance of D3 lymph node dissection because of the low-grade quality evidences of these studies and the low rate of apical lymph node metastasis [11, 21–25]. Although with a low metastatic rate, apical lymph node metastasis is an important prognostic factor for rectal cancer patients [8–11]. Furthermore, the rate of apical lymph node metastasis in rectal cancer is higher than that in the sigmoid colon [26]. Even skip metastases may also occur in distal colorectal cancer [8]. In our study, only 3 patients have apical lymph node metastasis. Anastomotic leakage is one of the most serious complications after anterior resection for rectal cancer. As we know, anastomotic perfusion and tension are the two most crucial risk factors for anastomotic leakage. Anastomotic tension could be easily identified during operation and solved by mobilizing the splenic flexure. However, it is difficult to identify the insufficient perfusion of anastomosis during operation. Although the indocyanine Green (ICG) technique [27–29] has recently been used to evaluate perfusion of anastomosis during operation, the accuracy is still insufficient. Blood flow of the proximal bowel and the anastomosis could be undoubtedly improved by LCA preservation in anatomy, some studies have also confirmed this [5–7]. ASCRS guideline [30] even emphasizes that HT may increase the risk of anastomotic leakage. However, some other studies [12–15] have suggested that LT has very little effect on reducing postoperative anastomotic leakage. A weak marginal artery or anatomically vulnerable to ischemia, such as Griffith’s point, and Sudeck’s critical point may be possible reasons. But these defects could be remedied by the preservation of the SA, compared with the preservation of LCA, it would obviously increase blood flow of the proximal bowel and reduce the incidence of related events, especially for a rectal cancer patient who received neoadjuvant therapy. On the other hand, extended lymphadenectomy (apical lymph node dissection) could undoubtedly achieve a better oncological outcome.

D3 lymph node dissection with more branches of the IMA preservation should be an optimal strategy at present. Therefore, we first propose a D3 lymph node dissection with LCA and first SA preservation in rectal cancer surgery. This technique combines the advantages of HT and LT, it gives consideration to oncological and functional outcomes. Recent research also shows it could reduce the incidence of anastomotic leakage without compromising oncological outcomes compare with D3 lymph node dissection with only LCA preservation. It is worth mentioning that all 68 patients with the LCA and first SA preservation before propensity score matching have no anastomotic leakage. In addition, we need to point out that not all patients are suitable for D3 lymph node dissection with LCA and first SA preservation, sometimes, preservation of first SA may lead to anastomotic tension, even lead to anastomotic failure. If the tension is identified after anastomosis, the patients would accept additional mobilization or even complete mobilization of the splenic flexure, sacrificing preserved LCA and/or SA is our last resort, and if the blood supply becomes weak, an ileostomy is a solution. Besides, this procedure is not suitable for a thin IMA and its branches, and it is also not suitable for a type IV IMA. And the dissection of the intermediate lymph nodes (LND2) is indeed a challenge of this technique. Last, our research is a retrospective study, so the possibility of selection bias is inevitable.

Ensuring radical resection of rectal cancer and preserving the blood supply of the anastomosis as much as possible are the pursuit of colorectal surgeons. Over the past 20 years, surgical techniques have improved significantly with the help of surgical equipment. It is believed that surgical technology will enter the era of refinement and individualization in the future. And our procedure happens to be a kind of refined and individualized rectal cancer surgery.

## Supplementary Information

**Additional file 1.**

## Data Availability

All data used during the study are available from the corresponding author by request.
